# Surface modification of PdlLGA microspheres with gelatine methacrylate: Evaluation of adsorption, entrapment, and oxygen plasma treatment approaches

**DOI:** 10.1016/j.actbio.2017.01.042

**Published:** 2017-04-15

**Authors:** Abdulrahman Baki, Cheryl V. Rahman, Lisa J. White, David J. Scurr, Omar Qutachi, Kevin M. Shakesheff

**Affiliations:** a Division of Drug Delivery and Tissue Engineering, School of Pharmacy, University of Nottingham, University Park, Nottingham NG7 2RD, UK; b Laboratory of Biophysics and Surface Analysis, School of Pharmacy, University of Nottingham, University Park, Nottingham NG7 2RD, UK

**Keywords:** Surface modification, PdlLGA microspheres, Gelatine methacrylate, Drug delivery, Tissue engineering

## Abstract

Injectable poly (dl-lactic-co-glycolic acid) (PdlLGA) microspheres are promising candidates as biodegradable controlled release carriers for drug and cell delivery applications; however, they have limited functional groups on the surface to enable dense grafting of tissue specific biocompatible molecules. In this study we have evaluated surface adsorption, entrapment and oxygen plasma treatment as three approaches to modify the surfaces of PdlLGA microspheres with gelatine methacrylate (gel-MA) as a biocompatible and photo cross-linkable macromolecule. Time of flight secondary ion mass spectroscopy (TOF SIMS) and X-ray photoelectron spectroscopy (XPS) were used to detect and quantify gel-MA on the surfaces. Fluorescent and scanning electron microscopies (SEM) were used to image the topographical changes. Human mesenchymal stem cells (hMSCs) of immortalised cell line were cultured on the surface of gel-MA modified PdlLGA microspheres and Presto-Blue assay was used to study the effect of different surface modifications on cell proliferation. Data analysis showed that the oxygen plasma treatment approach resulted in the highest density of gel-MA deposition. This study supports oxygen plasma treatment as a facile approach to modify the surface of injectable PdlLGA microspheres with macromolecules such as gel-MA to enhance proliferation rate of injected cells and potentially enable further grafting of tissue specific molecules.

**Statement of Significance:**

Poly (dl lactic-co-glycolic) acid (PdlLGA) microspheres offer limited functional groups on their surface to enable proper grafting of tissue specific bioactive molecules. To overcome this limitation, previous approaches have suggested using alkaline solutions to introduce active groups to the surface; however, they may compromise surface topography and lose any potential surface patterns. Plasma polymerisation of bioactive monomers has been suggested to enhance surface biocompatibility; however, it is not applicable on low vapour pressure macromolecules such as most extracellular matrix (ECM) proteins and growth factors. This study aims to evaluate three different approaches to modify the surface of PdlLGA microspheres with gelatine-methacrylate (gel-MA) to enable further grafting of cross-linkable biomolecules without compromising the surface topography or the biocompatibility of the system.

## Graphical abstract

Schematic figure showing the methods used to prepare Fit-C labelled gel-MA and the different surface treatment approaches used to modify the surface of PdlLGA microspheres with it.

**Figure:**
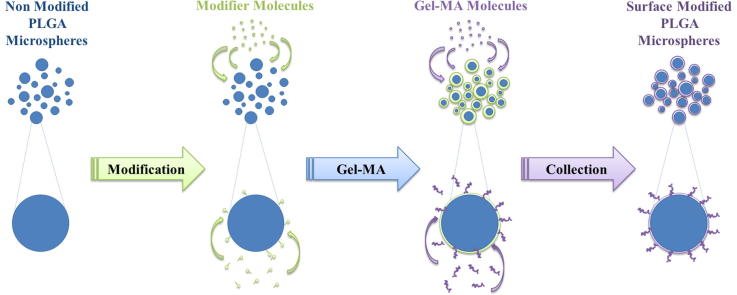

## 1 Introduction

Injectable PdlLGA microspheres have been proposed as cell delivery systems as they can form porous scaffolds [1], [2], [3] and control the release of signalling molecules into the cell niche [4], [5], [6], [7]. However, PdlLGA microspheres have limited reactive groups, such as carboxyl groups, available on the surface; this may limit the potential for further grafting of molecules required to support cell attachment and proliferation [8], [9]. To overcome this limitation, different approaches have been proposed to functionalise the surface and to enable further grafting [10], [11].

Some approaches have been reported to modify the surface by incorporating or copolymerising specific biomolecules such as collagen or biotinylated poly ethylene glycol (PEG) with the bulk polymer or monomer units conforming polymer chains [12], [13]. However, as these approaches may compromise the physiochemical properties of the bulk polymer, subsequent approaches focused on the surface modification instead [14]. Both surface adsorption and layer by layer deposition (LBL) of bioactive molecules used the electrostatic charges on the polymer surface to introduce oppositely charged molecules without changing polymer structure; however, these methods require a prior surface activation step to increase the surface charge [15], [16], [17], [18].

To increase the available surface charge, wet chemistry approaches have been suggested to functionalise the polymer surface by breaking polymer chains using alkaline solutions [19], [20], [21]; however, these approaches can also degrade the polymer surface and change surface topography [22]. Surface entrapment of molecules can functionalise inert polymer surfaces without altering their bulk chemistry [23]. This approach involves inducing polymer swelling with a modifying mixture made of a swelling agent [24] or a diluted solvent [25] with a dissolved modifying molecule. On the collapse of the swollen layer, the modifying molecule is trapped as a penetrating network at the surface of the polymer structure [26], [27], [28]. Electron beam irradiation and plasma induced polymerisation were reported to functionalise the surface with a minor effect on surface topography [29], [30], [31], [32], [33], [34], [35], [36], [37].

Gelatine has been used as cell delivery carrier and as a collagen derived molecule to enhance cell adhesion and proliferation [38], [39], [40]. Grafting methacrylate groups to the chains of macromolecules such as gelatine and gellan gum produces methacrylated chains such as gelatine methacrylate (gel-MA) and gellan gum methacrylate (GG-MA) which can be chemically crosslinked to obtain stiffer hydrogels with controllable degradation rate and mechanical properties [41], [42], [43], [44]. Gel-MA has also multiple cross-linkable sites which can be exploited for chemical grafting of other molecules for different tissue engineering applications [45].

Functionalising the polymer surface with cross-linkable small molecules such as vinyl based monomers was also reported to enable grafting of cross-linkable macromolecules. However, this approach may involve multistep reactions with corrosive chemicals to enable grafting which may compromise the activity of the grafted macromolecules and affect the topography of polymer surface [46], [47].

In this study, surface adsorption, surface entrapment, and oxygen plasma treatment approaches were investigated as single step approaches to modify the surface of PdlLGA microspheres with gel-MA macromolecules. The proposed surface modification approaches were later evaluated using different surface imaging and analytical techniques to determine the highest density of gel-MA molecules on the modified surface. Gel-MA modified PdlLGA microspheres were proposed to enhance stem cell interaction and to enable further grafting of further gel-MA molecules for different stem cell therapy applications.

## 2 Materials and methods

### 2.1 Material Preparation and fabrication

Poly (dl lactic-co-glycolic acid) (uncapped PdlLGA 8515, MW 52 KDa, Evonik – USA) microspheres were prepared with a preparation method reported earlier [7]. Briefly, 20% (w/v) PdlLGA solution in dichloromethane (DCM, Thermofisher Scientific, UK) was poured into 0.3% (w/v) poly(vinyl alcohol) (PVA, 86–89% hydrolysed Alfa Aesar-USA) solution in demineralised water and homogenised at 3000 RPM for 2 min using a propeller homogeniser (Silverson L5 M, UK). The resultant (o/w) emulsion was left stirring overnight to allow DCM solvent evaporation. PdlLGA microspheres were then centrifuged (MSE Mistral 1000, UK) and washed twice with distilled water, freeze-dried for 48 h (ModulyoD, Thermofisher Scientific, USA) and stored in a vacuum packed containers at (-20 °C).

Gelatine methacrylate (Gel-MA) was prepared following a published method [41]. Briefly, 10% gelatine (Type B gelatin from Bovine skin, Sigma-UK) solution in phosphate buffered saline (PBS Gibco, Thermofisher Scientific-UK) was prepared at 60 °C and kept under vigorous stirring (∼600 rpm, Thermofisher Scientific-UK) while 20 mL of methacrylic anhydride (MAA, Sigma-Germany) was injected dropwise over 20 min. The reaction mixture stirred at 60 °C for a further two hours until a white viscous liquid formed. The reaction was then stopped using 500 mL of warm PBS and the mixture was moved into dialysis tubes (Biodesign Dialysis Tubing D118, cut-off at 8000 MWCO, 30.5 cm length * 49.5 cm wet diameter, UK) and dialysed against daily changed distilled water for one week. After dialysis, the mixture became a clear to pale yellow solution. Gel-MA solution was later lyophilised to obtain white porous foam of gel-MA and stored at (−80 °C).

Fluorescein isothiocyanate (Fit-C isomer 1, Sigma-USA) was used to prepare Fit-C conjugated gel-MA as per manufacturer protocol to allow fluorescent detection of gel-MA layer on surface modified PdlLGA microspheres. Briefly, 80 mg of sodium hydroxide (NaOH, Sigma-UK) and 168 mg of sodium bicarbonate (NaHCO_3_, Sigma-UK) were dissolved in 20 mL of distilled water to obtain 1 N carbonate buffer solution with pH adjusted at 9.6 (Mettler Toledo – UK). Labelling solution was prepared by dissolving 10 mg of Fit-C in 1 mL of dimethyl sulfoxide (DMSO, Fischer Scientific-USA) in the dark. A small amount of gel-MA (500 mg) was dissolved in the freshly prepared carbonate buffer solution and the conjugation reaction was started by adding Fit-C solution dropwise in the dark with continuous stirring (∼300 rpm) to prevent Fit-C precipitation. Once all the fit-C solution was added, the mixture was incubated at 4 °C for 24 h in the dark with gentle stirring (∼200 rpm) to allow Fit-C conjugation to gel-MA free amine groups. The conjugation reaction was later neutralised by adding 20 mL of ammonium chloride (NH_4_Cl 1 N – Sigma, USA) to obtain a solution with (pH = 7). The final mixture was later dialysed (8000 MW cut-off dialysis bags) against distilled water to filter out unconjugated fit-C, freeze-dried, and stored at −80 °C (Supplementary Data 1).

### 2.2 Surface modification of PdlLGA microspheres

#### 2.2.1 Surface adsorption

**Fig. 1:**
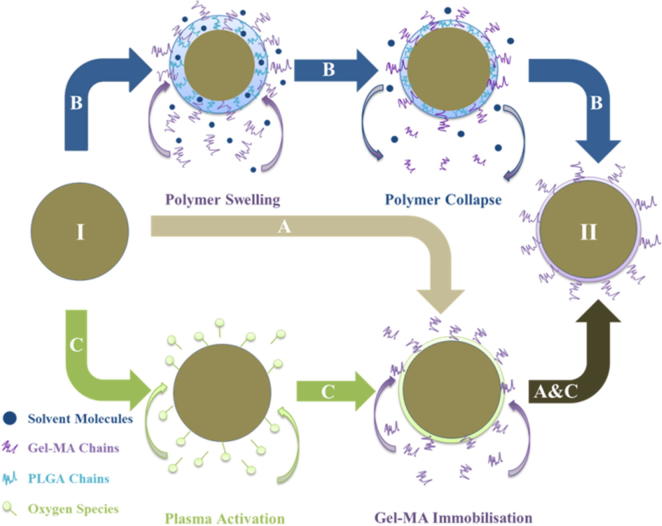
Schematic figure showing the different surface treatment approaches used to modify the surface of PdlLGA microspheres (I) with gel-MA to obtain gel-MA modified microspheres (II). Adsorption (A) is used to utilise the potential charge on the polymer chains to enable direct deposition of gel-MA molecules on the surface. Surface entrapment (B) is proposed to entrap gel-MA molecules on the surface of PdlLGA microspheres without compromising polymer chain composition. Finally, plasma treatment is proposed to immobilise gel-MA molecules on the surface of PdlLGA microspheres by introducing reactive oxygen species to PdlLGA polymer chains on the surface (C).

PdlLGA microspheres (500 mg) were dispersed in 2 mL of 10% (w/v) gel-MA /phosphate buffered saline (PBS) solution. The mixture was stirred at 300 rpm for 24 h before washing twice with water for collection as shown in provided schematic (Fig. 1).

#### 2.2.2 Surface entrapment

A modifying mixture was freshly prepared by mixing 0.75 mL of 2,2,2-trifluoroethanol (TFE, Sigma-UK) with 4.25 mL of 10% (w/v) gel-MA/PBS solution and PdlLGA microspheres (500 mg) were dispersed in 2 mL of the modifying mixture and kept under continuous stirring (∼300 rpm) to induce swelling and allow sufficient time for gel-MA to infiltrate the PdlLGA polymer matrix. After 24 h, the mixture was quenched with 20 mL of distilled water and kept under continuous stirring for further 30 min to induce the collapse of the swollen polymer surface and entrap gel-MA molecules. Treated microspheres were washed twice with water, freeze-dried, and stored at −20 °C for later analysis (Fig. 1).

#### 2.2.3 Plasma treatment

Low pressure oxygen plasma treatment was performed to introduce oxygen species to the surface. Briefly, PdlLGA microspheres (500 mg) were put in 25 mL glass vials in a plasma machine chamber (Diener Nano Type F, Diener Electronic GMBH – Germany) and kept rotating at 60 rpm using a rotary holder. Chamber pressure was pumped down to 20 mbar then oxygen gas was pumped into the chamber for 2 min to obtain a working pressure of 50 mbar. As working pressure was maintained at 50 mbar with continuous oxygen gas supply, plasma activation was initiated at 150 W (low frequency generator – 40 kHz) for 5 min. Following plasma activation, the chamber was purged with nitrogen gas for 2 min to dispose reactive oxygen ions and then air vented to atmospheric pressure to collect the samples. Following plasma treatment, PdlLGA microspheres were dispersed in 2 mL of 10% (w/v) gel-MA/PBS solution and kept stirring for 24 h to allow enough time for surface modification. Treated microspheres were later washed twice with water, freeze-dried, and stored at −20 °C for later analysis (Fig. 1).

### 2.3 Surface analysis of modified PdlLGA microspheres

#### 2.3.1 X-ray photoelectron spectroscopy (XPS)

XPS (Kratos Axis ULTRA DLD) with a monochromatic X-ray source of aluminium (Al Kα) with emission at (1486.6 eV) was used to detect and quantify gel-MA concentration on the surface of modified PdlLGA microspheres. Microspheres were mounted on carbon discs and both wide and high resolution scans were performed on three different areas of 0.25 × 0.25 mm size at room temperature while obtained peaks were analysed to quantify gel-MA related (N1s) peaks on the surface of modified microspheres using Casa XPS program (version 2.3.16 PR 1.6, Casa Software Ltd.).

#### 2.3.2 Time of flight secondary ion mass spectroscopy (ToF SIMS)

The ToF-SIMs instrument (ION-TOF, ION-TOF GmbH) was used to analyse treated PdlLGA microspheres using a bismuth (Bi^+^) ion source at room temperature. Three different areas of 0.25 × 0.25 mm size were scanned in each sample to analyse chemical composition of modified microsphere surface. Negative spectrums were collected and processed using a program provided by ION-TOF GmbH. ToF-SIMS spectra were analysed to create images of gel-MA distribution on the surface of modified PdlLGA microspheres.

### 2.4 Preparation of cross-sectioned PdlLGA microspheres

PdlLGA microspheres were mixed with 1 mL of O.C.T compound (Mounting Medium for Cryotomy, VWR international – Belgium) and moulded into aluminium sheet cylinders of 10 × 20 × 20 mm. The moulds were later immersed in N-Isopentane bath (Sigma-UK) frozen in liquid nitrogen until the O.C.T became solid white. Frozen moulds were mounted and cross-sectioned at −20 °C using a micro-cryotome (Leica CM1100 – Germany) to obtain 20 μm thickness slices of the O.C.T/microsphere mixture. Slices containing cross-sectioned microspheres were washed twice with distilled water to remove residual O.C.T, dispersed on a glass slide, and dried using a blast of N2 gas to obtain final microsphere cross-sections.

### 2.5 Imaging of surface modified PdlLGA microspheres

#### 2.5.1 Fluorescent microscopy imaging

Cross-sectioned PdlLGA microspheres modified with Fit-C labelled gel-MA were visualised on a Leica inverted fluorescent microscope (Leica DM IRB, Germany) using Fit-C light filter cube set at 495 nm. Images were taken using a microscope mounted camera (Qicam imaging, Canada) at a gain of 1 and a camera exposure time of 500 ms.

#### 2.5.2 Focused ion beam scanning electron microscopy (FIB-SEM)

Surface modified PdlLGA microspheres were mounted on carbon discs and coated with platinum for 5 min in a chamber attached to the FIB SEM (FEI Quanta 200 3D Dual Beam FIB-SEM). A focused ion beam was emitted on one of the PdlLGA microspheres to mill through the surface and to obtain a square hole exposing the inner structure of the microsphere. The FIB-SEM stage was later tilted to (30°) degree to enable imaging of the inner structure of PdlLGA microspheres using an electron beam acceleration of 15 kV.

To image cross-sectioned PdlLGA microspheres, samples were dispersed on carbon discs mounted on aluminium stubs (Agar, UK) and gold coated with benchtop plasma coater for 5 min at 25 mA (Leica EM SCD005 Sputter Coater) and imaged with SEM (JEOL 6060LV variable pressure SEM, UK) at electron beam acceleration of 14 kV and a working distance of 25 mm between electron beam source and sample surface.

### 2.6 Mesenchymal stem cell proliferation study

#### 2.6.1 Bicinchoninic acid (BCA) assay

Bicinchoninic acid assay (Micro BCA Protein Assay Kit, ThermoFisher Scientific-UK) was used as per manufacturer’s protocol to quantify the total content of gel-MA on the surface of PdlLGA microspheres.. Briefly, PdlLGA microspheres (100 mg) were dispersed in 1 mL of PBS and mixed with 1 mL of BCA mixture then incubated at 37 °C for 2 h. The mixture was injected through 0.2 μm mesh size filter to obtain clear PdlLGA microsphere-free solution. The solution was pipetted into 5 wells of a 96 well plate (Transparent 96 well plate, Falcon-USA) with 300 μL pipetted into each well. A plate reader (Tecan 200, Switzerland) was used to read the absorbance value of UV light at 562 nm to determine colour intensity. Intensity values were normalised against a standard curve of known gel-MA concentrations.

#### 2.6.2 Mesenchymal stem cell seeding and proliferation

A cell culture medium was prepared using Dulbecco’s Modified Eagle Medium (Gibco DMEM, ThermoFisher Scientific, UK) supplemented with (10% v/v) of foetal calf serum (ThermoFisher Scientific, UK), (1% v/v) of penicillin/streptomycin solution (anti-biotic/anti-mycotic 1x Gibco, ThermoFisher Scientific, UK), (1%v/v) of l-Glutamine (2 mM Gibco, ThermoFisher Scientific, UK), and (1% v/v) of MEM non-essential amino acids solution (Sigma-Aldrich, UK). Immortalised human mesenchymal stem cells obtained from the bone marrow of a healthy female donor (commercial cell, JCRB cell bank, Japan) were cultured and passaged 4 times, and detached using trypsin EDTA (Sigma Aldrich, UK) [48]. Cells were counted and centrifuged at 180*g* for 5 min, suspended in a freshly prepared cell culture medium at a cell density of 100,000 cells /mL and seeded onto 10 mg of PdlLGA microspheres at a seeding density of 10,000 cells/mg microspheres per well (non-tissue culture plate, Falcon, USA). Plates were then gently shaken to disperse cells, and incubated in a humidified tissue-culture incubator at 37 °C with 5% CO_2_.

The Presto-Blue cell viability assay (Invitrogen Life Sciences, UK) was used as per manufacturer protocol to assess cell viability and proliferation for different time points over 3 days post seeding on PdlLGA microspheres. Cultured cells were aspirated 4 h post seeding to wash away any non-attached cells on the microspheres (day 0), and Presto-Blue assay was performed on day 0, 1, and 3 to evaluate cell adhesion and proliferation on each sample. Briefly, Presto-Blue mixture was prepared as 1:9 parts of Presto-Blue solution: cell culture medium and each sample was incubated in 1 mL of the previous mixture for 45 min at 37 °C. After 45 min, five 100 μl aliquots of each sample were pipetted in 5 wells (R = 5) of 96 well plates (Flat Bottom Black, Coaster-US) and read on a Tecan plate reader at excitation/emission wavelengths of 535 nm and 590 nm respectively. Samples were later aspirated to remove excess Presto Blue mixture and incubated again with 1 mL of cell culture medium at 37 °C with 5% CO_2_ for the next reading time point.

### 2.7 Study design and statistics

Statistical analysis has been performed using GraphPad Prism (Version 6) analysis software. All values recorded in the graphs are averages of 3 different samples ± standard deviation (SD) and differences among groups were determined by ANOVA Tukey-Kramer multiple comparisons test and were considered to be significantly different if p value is less than 0.05.

## 3 Results

### 3.1 Detection of gel-MA on the modified microspheres

**Fig. 2:**
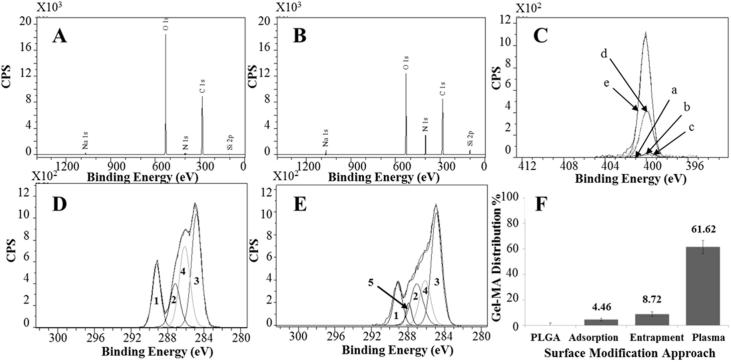
X-ray Spectroscopy (XPS) wide scan shows peak spectra of PdlLGA microspheres before (A) and after surface modification with gel-MA using plasma modification approach (B) where (N1s) peak from gel-MA peptide bonds can be seen at binding energy of ∼400 eV. A comparison between N1s peaks obtained from PdlLGA microspheres (C) shows the relative change in the intensity of N1s before (a), and after modification with gel-MA using adsorption (b), entrapment (c), and plasma approaches (d) compared to pure gel-MA (e). High resolution scan of C1s region from PdlLGA microspheres (D) shows three distinct peaks of PdlLGA (1,2, and 3) and a fourth peak of PVA (4), while a further gel-MA related peak (5) can be seen on plasma modified microspheres (E). Relative (N1s) peak area ratio of samples was normalised against (N1s) peak area of pure gel-MA films and used to quantify the concentration of gel-MA on the surface of gel-MA modified PdlLGA microspheres where a significant increase in the (N1s) area ratio can be seen on plasma and entrapment modified microspheres compared to adsorption and non-modified PdlLGA microspheres (F).

Wide XPS scan spectra included distinct peaks at C1s (∼285–289 eV) and O1s (∼531–533 eV) regions that are attributed to PdlLGA. A peak at N1 s region (∼398–402 eV) records the presence of a molecule with nitrogen containing bonds detected on gel-MA modified microspheres (Fig. 2). The different peak intensity of N1s regions on wide XPS scans between different surface modification approaches may indicate different densities of gel-MA on the surfaces (Supplementary Data 2). Quantification of the gel-MA related N1s peak showed an increase in the surface area of the N1s peak on the surface of plasma modified microspheres compared to adsorption and entrapment modified microspheres. As gel-MA is the only molecule containing nitrogen bonds, the increase in the surface area of N1s peaks indicate a respective increase in the density of gel-MA molecules on the surface (Table 1).

**Table 1:** Data analysis shows peak area distribution of different elements and their chemical states as obtained using XPS. Reported values represent averages of peak area percentage ± standard deviation obtained from wide XPS scans of three different scan areas.

Peak Area%	C1s	O1s	N1s
PdlLGA	70.13 ± 0.41	29.38 ± 0.65	–
Adsorption	70.3 ± 0.17	27.66 ± 0.47	0.23 ± 0.31
Entrapment	67.37 ± 0.96	31.44 ± 0.54	1.17 ± 0.36
Plasma	67.99 ± 0.83	22.39 ± 0.22	6.27 ± 0.9
Gel-MA	65.32 ± 1.11	20.55 ± 0.5	9.51 ± 0.11

High resolution scan analysis of the C1s region was performed on non-modified PdlLGA microspheres and gel-MA films to obtain the control peaks. Data obtained from non-modified PdlLGA microspheres were assigned to three PdlLGA peaks (C—C, C—O, and O=C—O) and one (C—O) poly vinyl alcohol (PVA) peak (1, 2, 3, and 4 peaks respectively – Fig. 2) as previously reported [49]. Control data from gel-MA films have shown three peaks at (∼286.3 eV), (∼288.1 eV), and (∼289.1 eV) assigned to (C—O), (N—C=O), and (O—C=O) (peak 6, 5, and 7 respectively) with allowed variability of peak position of ±0.1 eV and full width at half maximum values (FWHM) of ±0.2 eV (Supplementary Data 3). As gel-MA peaks (3) and (6) overlap PdlLGA and PVA peaks (3) and (4) respectively, the presence of the gel-MA peak (5-arrow) on C1s curves may indicate the presence of gel-MA on the surface of plasma modified microspheres (Fig. 2).

High resolution scan of the N1s region showed the increase in the intensity of the peak at ∼398 nm on gel-MA modified PdlLGA microspheres compared to non-modified PdlLGA microspheres (Supplementary Data 4). Different C1s peaks were assigned to relevant chemical states of carbon atoms found on the different amino acids found in gel-MA molecules (Supplementary Data 5). However, as most of gel-MA assigned C1s peaks overlap those of PdlLGA, the surface area of N1s peaks was used instead of that of C1s peaks to quantify gel-MA density on the surface.

**Fig. 3:**
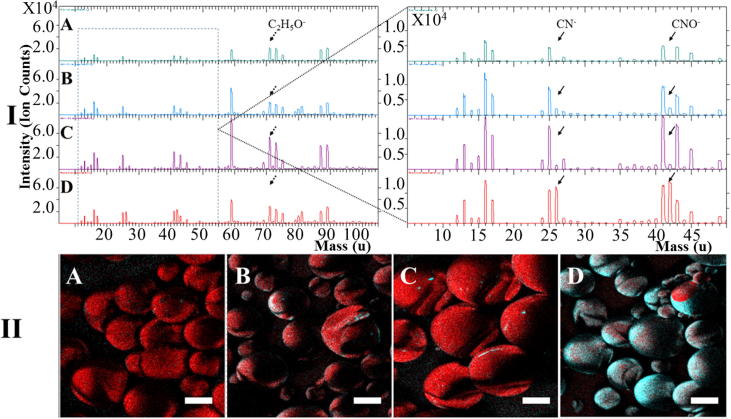
Time of Flight Secondary Ion Mass Spectrometry (ToF SIMS) ion peak spectra of PdlLGA microspheres before (A) and after modification with gel-MA using adsorption (B), entrapment (C), and plasma treatment approaches (D). Increasing ion counts of peptide bond related peaks (CN^−^) and (CNO^−^) can be seen on gel-MA modified microspheres (solid arrows) using adsorption, entrapment and plasma treatment approaches compared to (C_2_H_5_O^−^) PdlLGA ions (dotted arrow) respectively (I). Ion mapping images of (C_2_H_5_O^−^) and (CNO^−^) ions related to PdlLGA (red) and gel-MA (cyan) respectively, show homogenous distribution of gel-MA ions on the surface of plasma modified microspheres (D) compared to heterogeneous – or spotty – distribution on the surface of entrapment (C) and adsorption (B) modified microspheres (II). On the other hand, no clear sign of gel-MA specific ions can be seen on non-modified PdlLGA microspheres (A) (Scale bar = 50 μm).

Negative ion spectra of gel-MA modified PdlLGA microspheres have shown specific ion peaks at an ion mass of (26 *m*/*z*) and (42 *m*/*z*) which have been assigned to gel-MA peptide bond ions (CN^−^) and (CNO^−^), while specific PdlLGA ion peaks have been obtained from non-modified PdlLGA microspheres at (71 *m*/*z*) and assigned to (C_3_H_3_O_2_^−^) (Fig. 3-I). While non-modified PdlLGA microspheres have not shown a detectable number of gel-MA ions, an increase in the number of gel-MA specific ions has been observed on the surface of PdlLGA microspheres following gel-MA modification using plasma compared to less increase with entrapment and adsorption approaches. Images of gel-MA specific ion distribution on the surface of PdlLGA microspheres have shown a homogenous distribution of gel-MA ions on the surface of PdlLGA microspheres modified using plasma approach. On the other hand, PdlLGA microspheres modified using entrapment or adsorption approaches have shown a heterogeneous -or island like-distribution of gel-MA specific ions with limited surface coverage (Table 2) compared to no observable distribution on the surface of non-modified PdlLGA microspheres (Fig. 3-II). Other figures obtained from the optimisation process of both plasma and entrapment approaches can be found in the appendix (Supplementary Data 6, 7, and 8).

**Table 2:** Average surface coverage of gel-MA molecules on the surface of PdlLGA microspheres following modification with different surface modification approaches. Values are obtained from ToF SIMs image data analysis and correspond to the average coverage with gel-MA on the surface of 9 different microspheres ± standard deviation.

Surface Modification Approach	PLGA	Adsorption	Entrapment	Plasma
Gel-MA Surface Coverage%	0	15.7 ± 6.1	2.3 ± 0.8	89.3 ± 21.3

### 3.2 Imaging of modified PdlLGA microspheres

**Fig. 4:**
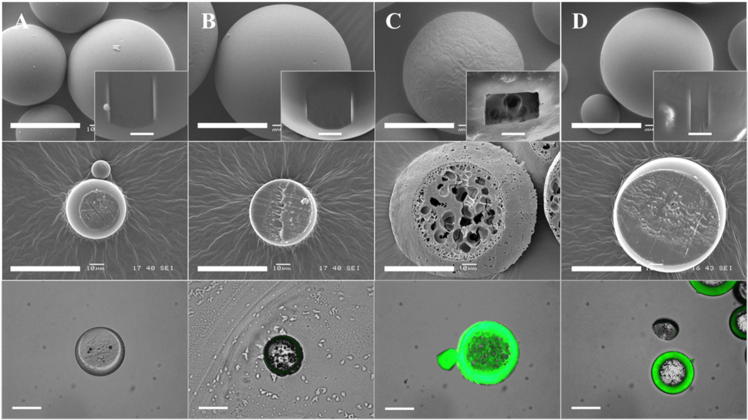
Scanning electron microscopy (SEM) images of PdlLGA microspheres before (A) and after modification with gel-MA using surface adsorption (B), surface entrapment (C), and oxygen plasma treatment approaches (D). Morphological changes can be observed on the surface of entrapment modified microspheres compared to a smooth round surface of all of the other conditions (Top raw). Further inspection of the morphological changes using a focused ion beam (FIB SEM) has revealed a porous structure underneath the surface of entrapment modified microspheres compared to solid fill inside all of the other conditions (magnified figures). Images of cross-sectioned PdlLGA microspheres (middle row) obtained with SEM show a porous structure with a decreasing pore size closer to the surface of entrapment modified microspheres while no observable changes have been noticed using other approaches. Fluorescent images of cross-sectioned microspheres (bottom row) show the depth of surface modification effect using Fit-C labelled gel-MA. While no clear presence of gel-MA on the non-modified (A) and adsorption modified PdlLGA microspheres (B), a considerable amount of Fit-C labelled gel-MA can be observed inside entrapment modified microspheres (C) compared to more surface specific localisation of fit-C labelled gel-MA on plasma modified microspheres (D) (Scale bar: all figures = 50 um, magnified figures = 5 um).

Surface structure of PdlLGA microspheres before and after modification with gel-MA was investigated using SEM. Microspheres modified using both adsorption and plasma modification approaches were spherical with smooth surfaces similar to the non-modified PdlLGA microspheres, while microspheres modified using the entrapment approach had a rougher surface with some evidence of porosity (Fig. 4-top row). Further probing of the structural changes using (FIB-SEM) showed no clear changes to the internal structures of PdlLGA microspheres before and after surface modification using both adsorption and plasma approaches (Fig. 4-magnified top). On the other hand, a porous internal structure was observed inside microspheres modified using the surface entrapment approach (Fig. 4-magnified top).

Further investigation of the internal structures using cross-sectioned PdlLGA microspheres revealed a highly porous internal structure underneath the surface following modification with the entrapment approach. The microspheres modified using adsorption and plasma approaches did not show any observable changes to internal structure compared to non-modified PdlLGA microspheres (Fig. 4-middle row).

Fluorescent microscope images showed a fluorescent layer on the outer surface of the cross-sectioned PdlLGA microspheres modified using both plasma and entrapment approaches, while a negligible fluorescent layer not detectable against the background fluorescence could be seen on the adsorption modified compared to non-modified PdlLGA microspheres where no observable fluorescent layer could be seen on the surface (Fig. 4-bottom row). Moreover, a fluorescent deposition could also be seen inside the entrapment modified microspheres compared to no fluorescent deposition inside plasma or adsorption modified microspheres (Supplementary Data 9).

### 3.3 Cell viability and proliferation

**Fig. 5:**
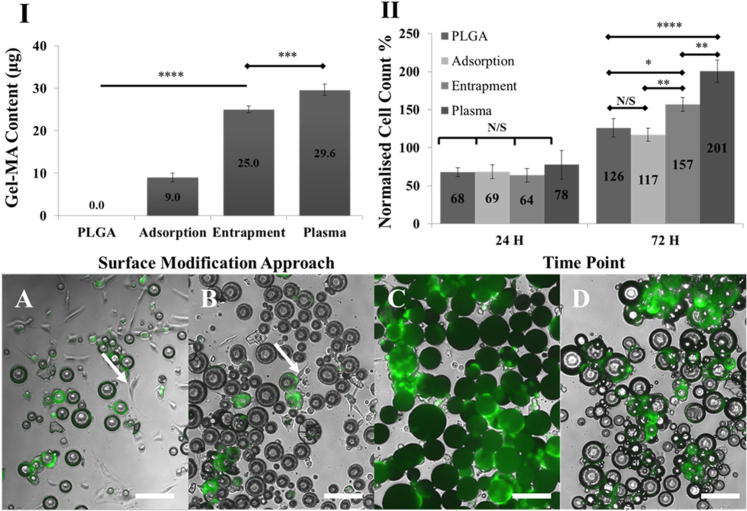
Quantification of gel-MA content on the surface of 100 mg of PdlLGA microspheres using BCA assay shows significant increase in gel-MA amount following modification with adsorption, entrapment, and plasma modification approaches respectively (I). Quantification of metabolic activity of immortalised human mesenchymal stem cells cultured on non-modified and gel-MA modified PdlLGA microspheres using Presto Blue assay shows significant increase in cell proliferation after 72 h of cell culture. All measurements are normalised to the activity measurements of cells cultured on a tissue culture treated plates over the same period of time. While no significant changes in cell metabolic activity can be noticed after 24 h; a significant increase in cell metabolic activity can be observed on plasma and entrapment modified PdlLGA microspheres compared to adsorption modified PdlLGA microspheres. Images taken after 72 h of cells cultured on microspheres show weak attachment of cells on both non-modified (A) and adsorption modified (B) PdlLGA microspheres where cells tend to grow on the substrate as well (arrows) while good cell attachment can be seen on both entrapment (C) and plasma modified PdlLGA microspheres (D). (n = 3, P Value is (^****^) <0.0001, (^***^) P < 0.001, (^**^) P < 0.01, (^*^) P < 0.05, and (ns) P > 0.05) (Scale Bar = 200 um).

BCA assay data normalised against readings from PdlLGA microspheres have shown an increase in the amount of gel-MA on the surface of plasma and entrapment modified PdlLGA microspheres compared to adsorption modified and non-modified PdlLGA microspheres respectively (P < 0.001) (Fig. 5-I). Immortalised hMSCs metabolic activity was measured using a Presto-Blue assay to study the effect of different surface modification approaches on cell adhesion and proliferation on the surfaces of PdlLGA microspheres over time. All Presto-Blue assay readings were normalised against readings from cells cultured on tissue culture plates. A cell survival study using a Presto-Blue assay over 72 h has shown that the immortalised hMSCs proliferation on gel-MA modified PdlLGA microspheres has not been compromised compared to non-modified PdlLGA microspheres. Presto-Blue assay readings after 24 h of cell culture did not show any significant change between different samples, while readings after 72 h showed a significant increase in both plasma and entrapment modified microspheres compared to adsorption modified and non-modified microspheres (Fig. 5-II). Microscope images showed a relatively higher number of cells growing on the surface of entrapment and plasma modified microspheres compared to both adsorption and non-modified microspheres. Cells were exclusively localised on the surface of plasma and entrapment modified microspheres, while they seemed to be non-specifically presented onto the surface of adsorption modified and non-modified PdlLGA microspheres as well as on the cell culture substrate (Fig. 5-A, B, C, and D).

## 4 Discussion

PdlLGA microspheres offer a potential injectable cell delivery system with controllable growth factor release for different tissue engineering applications [4], [5], [6], [7]. However, as with other polyester polymers, PdlLGA have limited functional groups on the surface which limit grafting of tissue specific bioactive molecules to control cell behaviour. This study has proposed modifying the surface of PdlLGA microspheres with gel-MA to enable grafting of further tissue specific biomolecules through the methacrylate group without compromising surface biocompatibility. Gel-MA molecules have the potential to form hydrogels with tunable elastic properties upon photo-crosslinking. Unlike gelatine, gel-MA molecules contain the methacrylate vinyl groups which can be used to further graft gel-MA or other bioactive molecules containing vinyl groups [43], [50]. Moreover, non-crosslinked gelatine hydrogels dissolve over 24 h of incubation at 37 °C while gel-MA hydrogels prepared using UV photo-crosslinking offer controllable elastic properties and do not dissolve over 24 h of incubation at 37 °C (Supplementary Data 11).

Terminally uncapped PdlLGA was used in this study as it was reported to have higher surface energy due to the presence of carboxyl end groups compared to the lower surface energy of terminally capped PdlLGA which has a methylated carboxyl end groups [51], [52], [53]. Surface entrapment was proposed in this study to evaluate the potential incorporation of gel-MA molecules within PdlLGA polymer network on the surface as previously suggested [54]. Likewise, oxygen plasma treatment was proposed in this study to improve the available oxygen species on the surface of PdlLGA microspheres and immobilise gel-MA molecules.

This study has demonstrated that PdlLGA microspheres modified using oxygen plasma treatment had higher density of gel-MA molecules on the surface compared to those modified using entrapment or adsorption approaches respectively. Since adsorption of gel-MA molecules would mainly depend on the available surface energy, the low density of deposited gel-MA molecules on the surface of adsorption modified microspheres indicate a low surface energy on the surface of uncapped PdlLGA microspheres.

On the other hand, as plasma induced oxygen species have been suggested to include peroxide, hydroxyl, and carboxylic groups [55], [56], [57]; the increase in gel-MA content following oxygen plasma modification could be attributed to these oxygen species as well. Peroxide groups have been reported to be chemically unstable and could potentially form reactive radicals which may attack electron rich vinyl groups and immobilise gel-MA molecules through covalent bonding. Unstable peroxide groups may also transform into hydroxyl or carboxyl groups which could increase surface energy and further enhance the deposition of gel-MA molecules on the surface [58].

The structural changes seen on the surface as well as inside the entrapment modified microspheres could be attributed to the partial restructuring of the PdlLGA network during the entrapment process. The prolonged exposure to solvent molecules enables deeper penetration and higher densities of modifying molecules to entrap on the polymer surface as previously suggested in literature [59]. This study has shown that prolonged exposure to the solvent can also induce porous structures underneath the surface.

Fluorescent microscope images have shown a fluorescent layer on the surface of entrapment and plasma modified microspheres only which indicates successful modification with fit-C gel-MA. No detectable fluorescent layer was observed on the adsorption modified microspheres which indicates little deposition of fit-C gel-MA on the surface. Moreover, the detection of fit-C gel-MA on the surface along with the porous structures inside the entrapment modified microspheres may indicate a modification not just on the surface, but deep inside the microspheres as well.

The density of gel-MA on the surface of entrapment modified microspheres was comparable to that on the adsorption modified microspheres as shown with XPS and ToF SIMs data. However, the overall content of gel-MA quantified with BCA assay on the entrapment modified microspheres was higher than that detected on the surface of adsorption modified microspheres. This may indicate that entrapment approach has increased the available surface area. As the BCA assay measures the total gel-MA content of microspheres in contact with the BCA mixture, the high surface area of the porous entrapment modified microspheres may potentially contain higher amounts of gel-MA. Therefore, the BCA mixture would react with a higher number of gel-MA molecules on the surface of entrapment modified microspheres compared to the relatively less gel-MA molecules found on the lower surface area of non-porous adsorption modified microspheres.

The low density of gel-MA molecules detected by XPS and ToF SIMs on the surface of entrapment modified microspheres may be attributed to the fact that both techniques are surface specific. Both ToF SIMs and XPS can probe few nanometres into the analysed surface [60] while fluorescent microscopy and BCA can detect the whole surface including the internal surfaces of the porous microspheres. This may explain the relatively higher gel-MA density detected with BCA and fluorescent microscopy on the surface of entrapment modified microspheres compared to the non-porous adsorption modified microspheres.

The significant increase in the metabolic activity of immortalised hMSCs cultured on the plasma and entrapment modified microspheres compared to the non-modified and adsorption modified microspheres can be attributed to the high density of gel-MA on the surface. On the other hand, the significant increase in the metabolic activity with the entrapment modified compared to the adsorption modified microspheres can be attributed to the high surface area of the porous entrapment modified microspheres which seems to introduce higher gel-MA domains for cell adhesion. This may also explain the localised growth of the immortalised hMSCs on the surface of plasma and entrapment modified microspheres compared to the non-specific growth on the cell culture substrates as well as on the surface of adsorption modified and non-modified microspheres where gel-MA content is negligible.

Finally, fit-C labelled gel-MA has been proposed to evaluate the potential grafting of crosslinkable macromolecules on the surface of gel-MA modified PdlLGA microspheres. ToF SIMs images of gelatine modified microspheres prepared using the plasma treatment approach showed homogenous distribution of gelatine molecules on the surface of PdlLGA microspheres (Supplementary Data 12). Grafting fit-C labelled gel-MA molecules on the surface of non-modified, gelatine, and gel-MA modified PdlLGA microspheres showed a fluorescent layer related to the fit-C gel-MA molecules on the surface of gel-MA modified microspheres only. In comparison, no detectable fluorescent layer could be seen on the surface of non-modified and gelatine modified PdlLGA microspheres indicating a negligible deposition of fit-C gel-MA. This can be explained with the presence of the methacrylate vinyl groups on the surface of gel-MA modified PdlLGA microspheres which can form covalent bonds with fit-C gel-MA molecules upon photo-grafting. In comparison, as both gelatine molecules and PdlLGA chains do not offer the crosslinkable vinyl groups, they would not enable grafting of fit-C gel-MA molecules (Supplementary Data 13).

## 5 Conclusion

This study has evaluated three different approaches to modify the surface of PdlLGA microspheres with gel-MA: surface adsorption, surface entrapment, and oxygen plasma treatment. Different surface analytical and quantitative assays have shown that the oxygen plasma treatment approach produced the highest density of gel-MA on the surface of modified PdlLGA microspheres followed by entrapment and adsorption approaches respectively. Moreover, different microscopy images have shown porous structures inside PdlLGA microspheres modified using surface entrapment approach. This may suggest a deep penetration of gel-MA molecules underneath the surface of microspheres and a possible restructuring of the polymer microsphere during the process. On the other hand, the significant increase in cell metabolic activity of immortalised hMSCs cultured for 72 h on the surface of plasma and entrapment modified microspheres compared to adsorption and non-modified microspheres can be related to the gel-MA content on the surface. This in turn would suggest no adverse effects of the proposed surface modification approaches on the biocompatibility of the system.

The modified microspheres could be utilised to induce further grafting of cross-linkable biomolecules to obtain tissue specific functionalities. Moreover, as gel-MA has been reported to produce hydrogels with tissue specific mechanical properties, gel-MA modified PdlLGA microspheres could potentially be further grafted with gel-MA molecules to produce hydrogel coated microspheres with tissue specific elastic properties.

## Figures and Tables

**Supplementary data 1:**
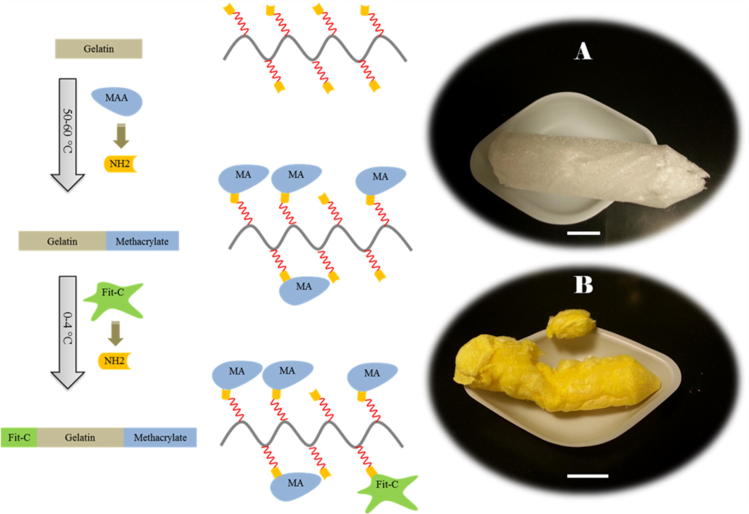
Schematic figure showing the method used to prepare Fit-C labelled Gel-MA and the appearance of gel-MA before (A) and after labelling with fit-C (B). (Scale bar = 2 cm).

**Supplementary data 2:**
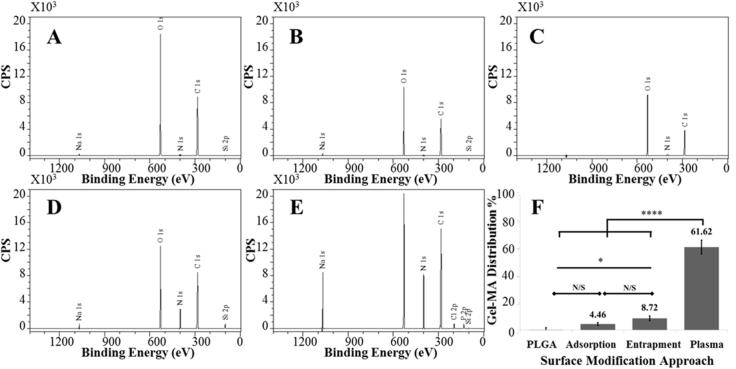
X-Ray Spectroscopy (XPS) wide scan shows peak spectra of PdlLGA microspheres before (A) and after surface modification with gel-MA using adsorption (B), entrapment (C), and plasma (D) modification approaches. Significant (N1s) peak from gel-MA peptide bonds can be seen on gel-MA (E) and on plasma modified microspheres. Relative (N1s) peak area ratio of samples was normalised against (N1s) peak area of pure gel-MA films and used to quantify the concentration of gel-MA on the surface of gel-MA modified PdlLGA microspheres where a significant increase in the (N1s) area ratio can be seen on plasma and entrapment modified microspheres compared to adsorption and non-modified PdlLGA microspheres (F).

**Supplementary data 3:**
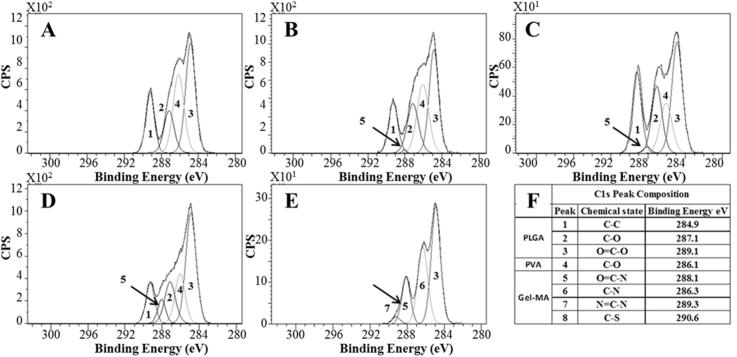
X-ray Photoelectron Spectroscopy (XPS) high resolution analysis of C1s regions shows curves from PdlLGA microspheres before (A) and after surface modification with gel-MA using adsorption (B), entrapment (C), and plasma (D) modification approaches. The curve obtained from Gel-MA films (E) shows five fitted peaks assigned to (N—C=O) at ∼288.1 (5), (C—N) at 286.3 eV (6), (N=C—N) at 289.3 eV (7), and a negligible peak (C—S) at 290.6 eV (8). Curves obtained from non-modified PdlLGA microspheres show three PdlLGA attributed peaks assigned to: (C—C & C—H) at ∼284.9 (1), (C—O) at ∼287.1 eV (2), and (O—C=O) at ∼289.1 eV (3), and a further PVA attributed peak assigned to (C—O) at ∼286.1 eV (4). The curves obtained from gel-MA modified PdlLGA microspheres show significant increase in the area of gel-MA related peaks (5) using plasma approach compared to entrapment and adsorption approaches.

**Supplementary data 4:**
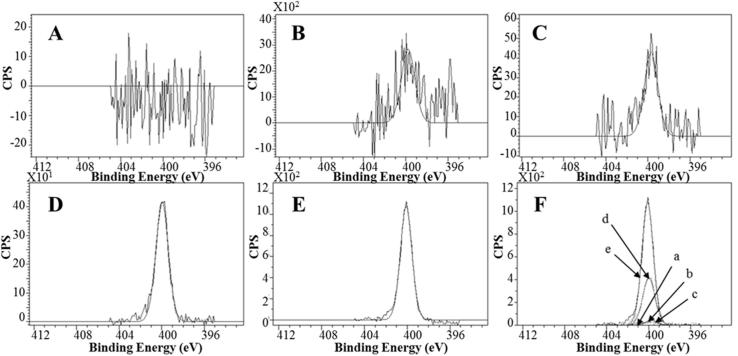
X-Ray Photoelectron Spectroscopy (XPS) high resolution analysis of N1s region shows curves from PdlLGA microspheres before (A) and after surface modification with gel-MA using adsorption (B), entrapment (C), and plasma (D) modification approaches. Unlike the curves obtained from Gel-MA films (E) and plasma modified microspheres where a significant peak at 400 eV can be observed; the entrapment and adsorption modified microspheres show a distorted peak compared to no clear peak on non-modified PdlLGA microspheres. An overlay of the samples N1s regions (F) shows the significance of peak areas from gel-MA films (e) and plasma (d) compared to entrapment (c), adsorption (b), and non-modified PdlLGA microspheres (a).

**Supplementary Data 5:**
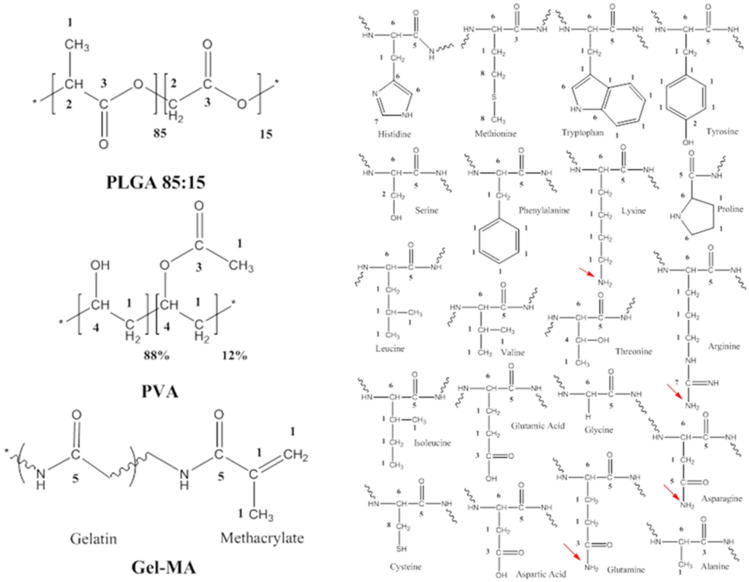
X-Ray Photoelectron Spectroscopy (XPS) assignment of C1s peaks for PdlLGA, PVA, and Gel-MA. Assignments for Gel-MA peaks are further explained with the amino-acids found in gel-MA molecules.

**Supplementary Data 6:**
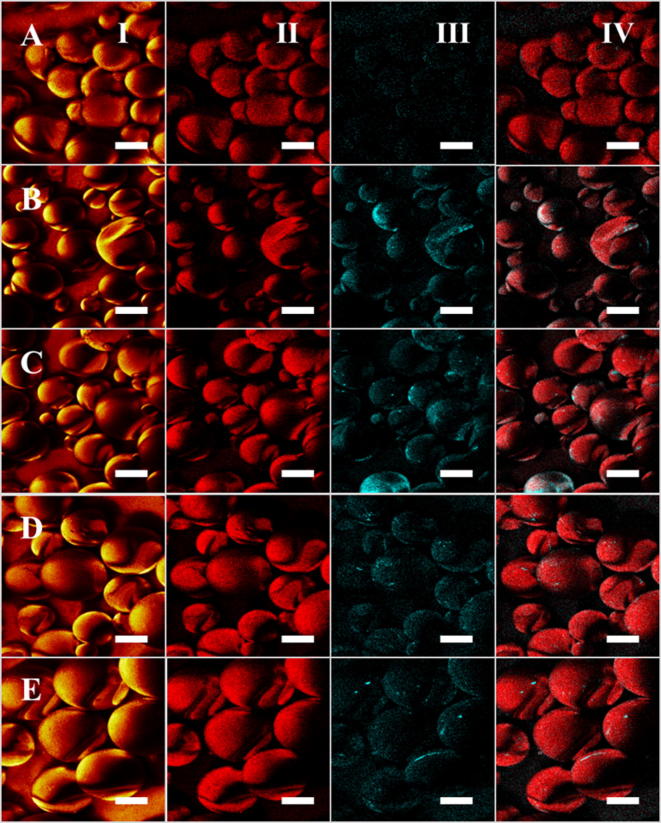
Ion mapping images of PdlLGA microspheres using time of flight secondary ion mass spectrometry (ToF SIMS) ion peak spectra of PdlLGA microspheres before (A) and after modification with gel-MA solution using different ratios of TFE to induce swelling and entrapment of gel-MA on the surface: (B) 0% (Adsorption approach), (C) 5%, (D) 10%, and (E) 15% . Images of full range of ions are shown on (I) while PdlLGA specific ions (C2H5O-) are pseudo-coloured red (II), gel-MA specific ions (CNO-) are pseudo-coloured cyan (III), and an overlaid image of PdlLGA and gel-MA ions are seen on (IV). Spotty distribution of gel-MA specific ions can be seen on gel-MA modified PLGA microspheres suggesting a low concentration of gel-MA on the surface following modification with adsorption and entrapment approaches.

**Supplementary Data 7:**
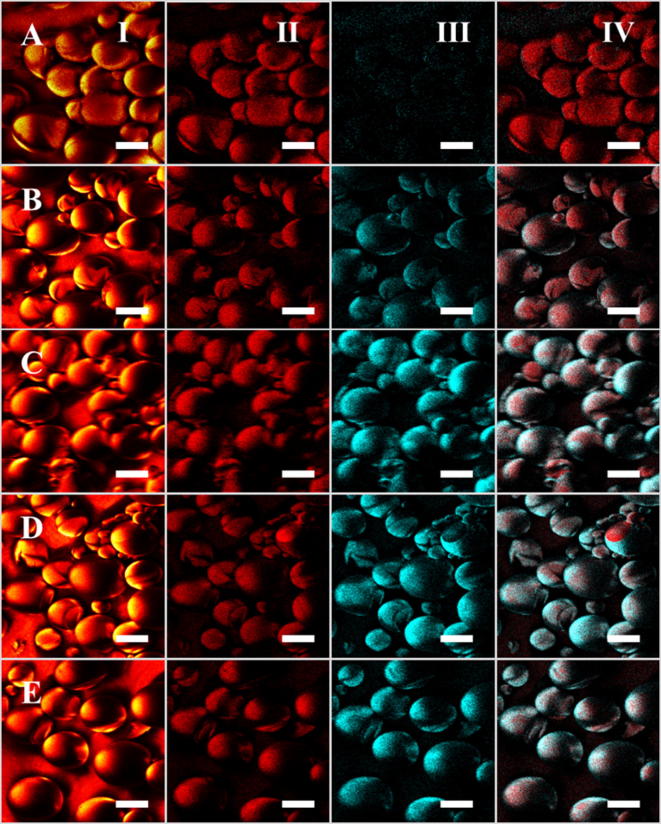
Ion mapping images of PdlLGA microspheres using time of flight secondary ion mass spectrometry (ToF SIMS) ion peak spectra of PdlLGA microspheres before (A) and after modification with gel-MA solution using different exposure times of oxygen plasma (B) 1.25 min, (C) 2.5 min, (D) 5 min, and (E) 10 min. Images of full range of ions are shown on (I) while PdlLGA specific ions (C2H5O−) are pseudo-coloured red (II), gel-MA specific ions (CNO-) are pseudo-coloured cyan (III), and an overlaid image of PdlLGA and gel-MA ions are seen on (IV). A gradual increase in gel-MA distribution on the surface of gel-MA modified PdlLGA microspheres can be observed following the gradual increase in the time of exposure to oxygen plasma.

**Supplementary Data 8:**
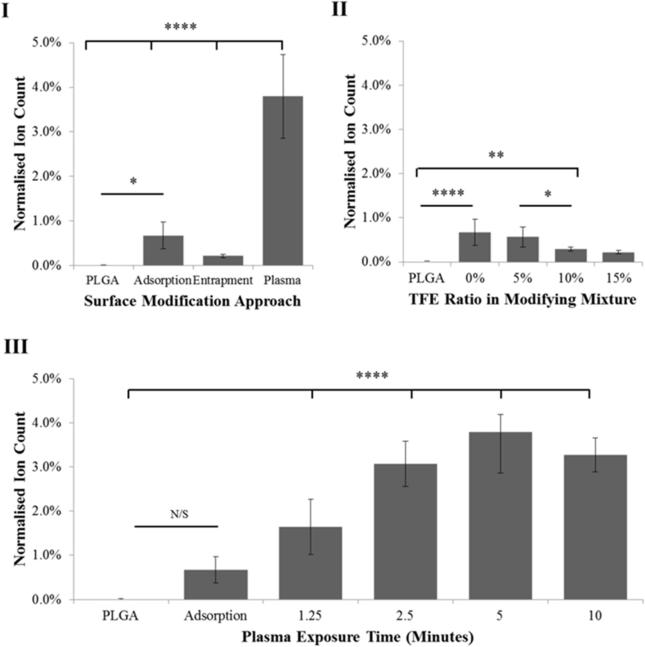
Quantification of ToF SIMs ion mapping images of PdlLGA microspheres shows the average value ± standard deviation (SD) of specific gel-MA ion count normalised against the total ion count from each microsphere out of nine microspheres per image. All graphs were normalised against normalised gel-MA ion count from non-modified PdlLGA microspheres to minimise the effect of background ions. (I) A comparison of ion counts from gel-MA modified microspheres show a significant increase in gel-MA ion count from plasma modified compared to adsorption, entrapment and non-modified PdlLGA microspheres (I). A comparison between different TFE ratios used in the surface entrapment approach (II) and different oxygen plasma exposure times (III) show the significant change in the normalised ion count of gel-MA specific ions compared to non-modified PdlLGA microspheres

**Supplementary Data 9:**
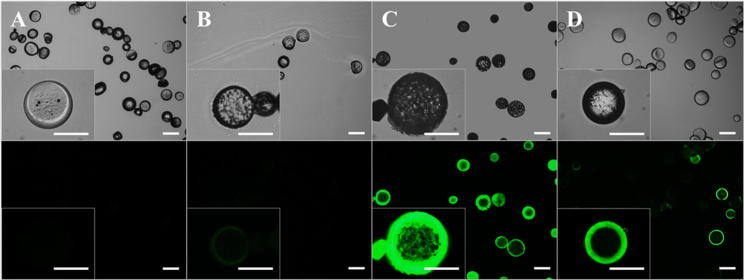
Images of PdlLGA microspheres taken following slicing with a cryo-microtome show the effect of modification approaches on the polymer bulk underneath the surface. Bright-field microscope images (Top) of PdlLGA microspheres before (A) and after modification with gel-MA using surface adsorption (B), entrapment (C), and plasma treatment approaches (D). Fluorescent images of cross-sectioned microspheres (bottom) show the depth of surface modification effect using Fit-C labelled gel-MA. Considerable amount of Fit-C gel-MA can be observed inside entrapment modified microspheres (C) compared to more surface specific modification with plasma treated microspheres (D) and no clear presence of gel-MA on adsorption modified (B) and non-modified microspheres (A). (Scale bar = 100 μm).

**Supplementary Data 10:**
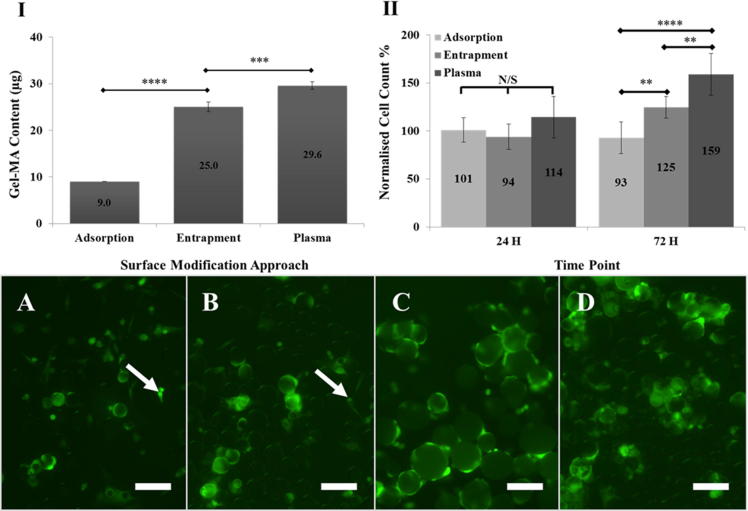
Quantification of gel-MA content on the surface of 100 mg of PdlLGA microspheres using BCA assay shows significant increase in gel-MA amount following modification with adsorption, entrapment, and plasma modification approaches respectively (I). Quantification of metabolic activity of immortalised human mesenchymal stem cells cultured on non-modified and gel-MA modified PdlLGA microspheres using Presto Blue assay shows significant increase in cell proliferation after 72 h of cell culture. All measurements are normalised to the activity measurements of cells cultured on a non-modified PdlLGA microspheres over the same period of time. While no significant changes in cell metabolic activity can be noticed after 24 h; a significant increase in cell metabolic activity can be observed on plasma and entrapment modified PdlLGA microspheres compared to adsorption modified PdlLGA microspheres. Images taken after 72 h of cells cultured on microspheres show weak attachment of cells on both non-modified (A) and adsorption modified (B) PdlLGA microspheres where cells tend to grow on the substrate as well (arrows) while good cell attachment can be seen on both entrapment (C) and plasma modified PdlLGA microspheres (D). (n = 3, P Value is (^∗∗∗∗^) <0.0001, (^∗∗∗^) P < 0.001, (^∗∗^) P < 0.01, (^∗^) P < 0.05, and (ns) P > 0.05). Scale Bar = 200 μm

**Supplementary Data 11:**
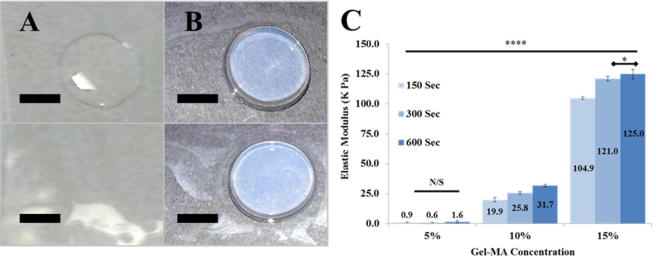
Images of gelatine (A) and gel-MA (B) discs prepared using 10% w/v solution containing 0.5% w/v Irgacure 2959 and UV photo-crosslinked for 5 min before (top) and after incubation at 24 h at 37°C (bottom). Elastic properties of gel-MA hydrogel discs prepared at different concentrations and UV exposure times show a significant change in the Young’s modulus value after 24 h of incubation at 37 °C upon altering gel-MA concentration in the solution or the UV exposure time (C). Elastic properties are obtained using compression test mode on the texture analyser with 2mm probe diameter and 1 cm2 hydrogel discs. Young’s modulus is calculated from the linear part of the stress strain curve relating to 0-10% strain ratio (Data corresponds to measurements obtained from 3 different discs of each sample (n = 3) and collected from 5 different positions of each disc (R=5) where statistically significant change is considered when P value obtained from one-way ANOVA test is (^∗∗∗∗^) <0.0001, and (^∗^) P < 0.05, while statistically insignificant when (ns) P > 0.05).

**Supplementary Data 12:**
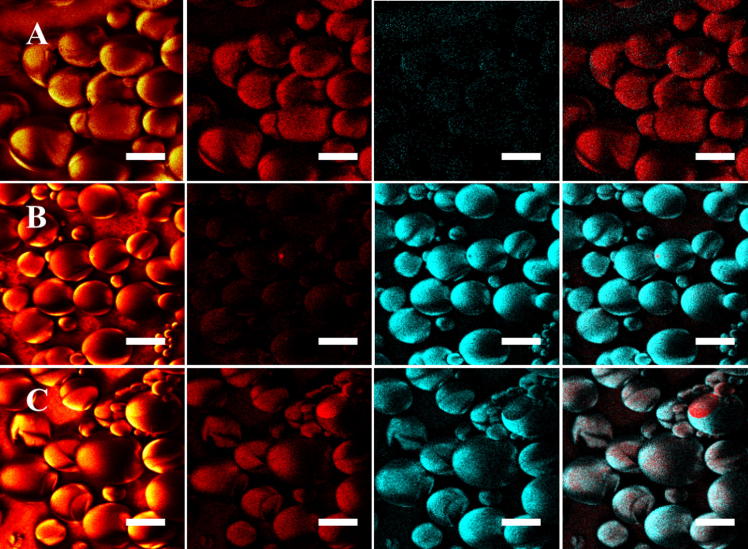
Ion mapping images of PdlLGA microspheres using time of flight secondary ion mass spectrometry (ToF SIMS) (left) where (C2H5O−) and (CNO−) ions correspond to PdlLGA (red) and gelatine (cyan) molecules respectively. Images show a homogenous distribution of gelatine ions on the surface of adsorption (B) and plasma modified microspheres (C) compared to negligible presence of gelatine specific ions on non-modified PdlLGA microspheres (A). (Scale bar = 50  μm)

**Supplementary Data 13:**
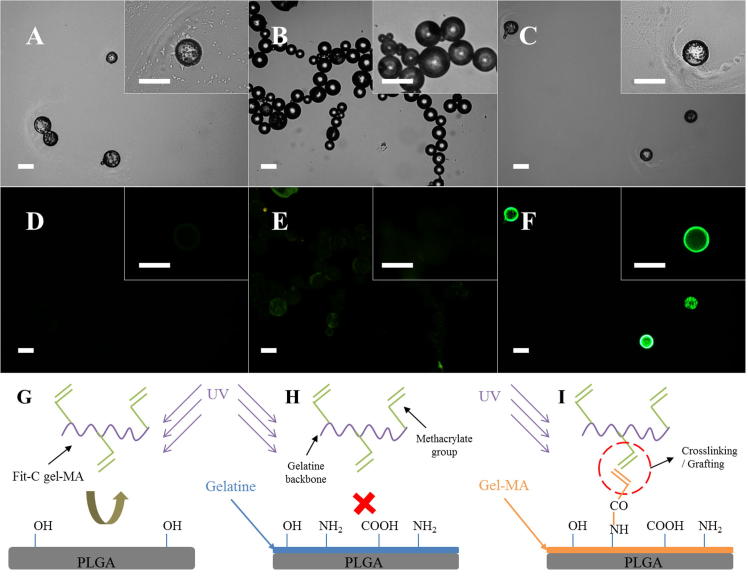
Bright field (top) and fluorescent images (bottom) of PdlLGA microspheres taken following grafting of fit-C labelled gel-MA on the surface of non-modified (A), gelatine modified (B), and gel-MA modified PdlLGA microspheres (C). Though bright-field microscope images do not show clear change in the morphology of different microspheres upon grafting, fluorescent images of the microspheres show a clear deposition of fit-C labelled gel-MA indicating successful grafting on the surface of gel-MA modified microspheres (C) compared to negligible deposition indicating poor grafting of fit-C labelled gel-MA on the surface of gelatine modified (B) and non-modified PdlLGA microspheres (A) respectively. Schematic overview of the grafting approach on the surface of PdlLGA microspheres shows the limited functional groups and the lack of crosslinking moieties on the surface of non-modified PdlLGA microspheres which prevents covalent grafting of gel-MA molecules (G). On the other hand, the hydrophilic surface of gelatine modified microspheres lacks the crosslinking methacrylate groups which may enable the adsorption but restricts the covalent grafting of fit-C gel-MA molecules on the surface (H). In comparison, gel-MA modified PdlLGA microspheres offer the methacrylate groups on the surface which can enable covalent grafting of fit-C gel-MA molecules through UV photo-crosslinking mechanism (I). (Scale bar = 100 μm)
